# Urea water bath: A novel treatment for monogenean parasite infections in hagfish (Agnatha: Myxiniformes: Myxinidae)

**DOI:** 10.1016/j.mex.2025.103518

**Published:** 2025-07-17

**Authors:** Taketeru Tomita, Hiroko Takaoka, Nozomi Hanahara

**Affiliations:** a Okinawa Churashima Research Institute, Okinawa Churashima Foundation, Motobu, Japan; b Okinawa Churaumi Aquarium, Okinawa Churashima Foundation, Motobu, Japan

**Keywords:** Aquarium, Artificial uterine fluid, Freshwater bath, Pathology, Salinity, Skin fluke

## Abstract

Monogenean flatworms are pathogenic parasites that infect the skin of marine fish and pose a major problem in aquaculture and aquarium industries. Freshwater baths are the most commonly used method to treat these infections. However, this method is not suitable for certain fish taxa, including hagfish (Agnatha: Myxiniformes: Myxinidae), which have low tolerance to hypoosmotic environments.

In the present study, we developed a treatment fluid specifically for use in hagfish. The fluid was prepared by dissolving urea in a mixture of chlorine-free tap water and seawater. A 5-min bath of three captive purple hagfish in the fluid successfully eliminated the parasites, with no apparent side effects. To date, this is the only known effective treatment for monogenean infections in hagfish.

• In this study, we developed a method to treat monogenean infections in hagfish.
• This method uses a fluid containing a high concentration of urea.
• Bathing infected hagfish in the fluid successfully eliminated the parasites without apparent side effects.

## Graphical abstract

**Figure:**
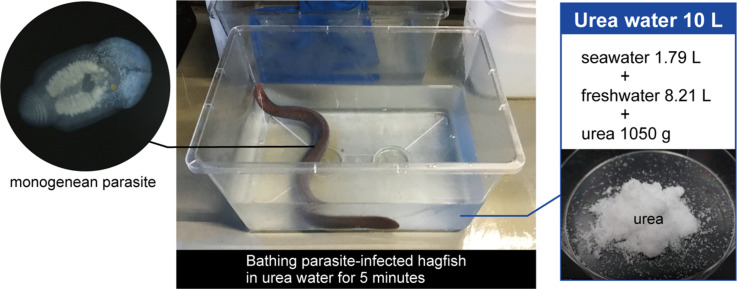

Specifications table

**Table:** 

**Subject area**	Veterinary Science and Veterinary Medicine
**More specific subject area**	Fish pathology
**Name of your method**	Urea water bathing
**Name and reference of original method**	Not applicable
**Resource availability**	Not applicable

## Background

Skin infections caused by parasitic flatworms in marine fishes have long been a major problem in the aquaculture and aquarium industries. The best-known examples of these parasites are capsalid monogeneans (including *Benedenia* spp. and *Neobenedenia* spp.). They comprise approximately 180 species and share a similar parasitic ecology, strongly attaching to the host’s skin surface with multiple pads and feeding on skin mucus and epithelial cells (reviewed in [1]). Infection leads to reduced growth rates, secondary bacterial infections, and increased host mortality. The high egg productivity of these parasites (e.g., >500 eggs/day in *B. seriolae* [2]) leads to rapid accumulation of larvae in captive environments, resulting in widespread infection outbreaks.

One effective treatment method for this condition in aquaculture is freshwater bathing. This involves placing the infected fish in freshwater for 3–5 min [3,4]. During this process, the parasites die due to their low tolerance to low-salinity conditions. This method offers advantages over drug administration, as it does not require specialized medical skills (e.g., injections) or costly chemicals.

Although freshwater baths are commonly used in aquaculture, their application in public aquaria is limited. This is because (1) the efficacy of the method has only been tested on a limited number of monogenean species commonly found in aquaculture, and it remains poorly evaluated for most monogenean species infecting fish in aquaria; and (2) freshwater tolerance among marine fishes varies widely [5], making freshwater baths unsuitable for certain taxa. Hagfish (Agnatha: Myxiniformes: Myxinidae) are extreme examples of fish with very low tolerance to hypoosmotic environments. Their gills are easily damaged by brief exposure to freshwater, and freshwater baths result in gill bleeding and death (HT, pers. obs.).

This study proposes a novel parasiticidal bathing fluid suitable for use in hagfish. This fluid contains a high concentration of urea and is characterized by high osmolality and low salinity. It is easy to prepare and cost-effective, making it suitable for treating monogenean infections in hagfish.

## Method details

### Construction of the fluid

A total of 10 L of urea-containing fluid (referred to as “urea water” in this study) was prepared by dissolving 1050 g of urea in a mixture of 8.21 L of chlorine-free tap water and 1.79 L of filtered seawater. The components of the fluid (i.e., urea, seawater, and tap water) were the same as those in the shark-specific “artificial uterine fluid (AUF)” developed in Tomita et al. [6], but the composition was greatly changed as follows: The urea concentration is three times of AUF; whereas the seawater concentration is one-third that of AUF. These changes result in a fluid with low salinity (approximately one-sixth that of seawater) and high osmotic pressure (approximately 1.7 times that of seawater). Chemical measurements of the fluid obtained using a DRI-CHEM NX600 automated clinical chemistry analyzer (Fujifilm Co., Tokyo, Japan) are shown in Table 1.

**Table 1:** Chemical measurements (*n* = 5) and osmotic pressure (*n* = 4) of urea water.

Measure	Unit	Average	*SD*
Sodium (Na)	mEq/L	89.8	1.8
Chlorine (Cl)	mEq/L	79.2	2.2
Urea Nitrogen (UN)	mg/dL	4375.4	102.0
Osmotic pressure	mmol/kg	1710.2	76.5

### Treatment protocol

**Fig. 1:**
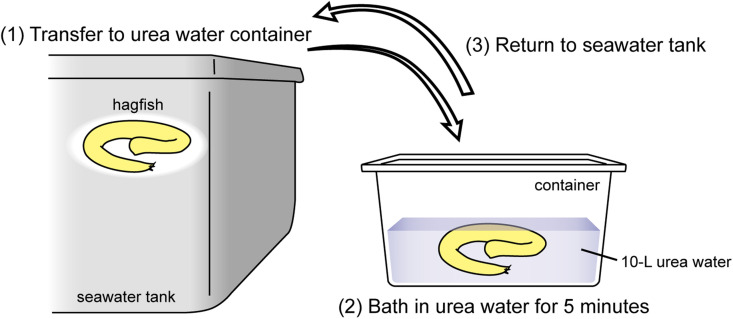
Protocol for the urea-water bath treatment in hagfish.

The infected fish were transported from the seawater tank to a container that includes 10-L of urea water. After bathing in urea water, the fish were returned to the seawater tank (Fig. 1).

## Method validation

### Evaluation of fluid chemistry and bathing duration

To evaluate the appropriate urea-water chemistry and bathing duration, we measured the time to mortality of monogenean parasites in different fluid compositions. We prepared four types of fluid with different chemical compositions: Fluid 1, which has the same chemistry as the shark-specific artificial uterine fluid (prepared by dissolving 350 g of urea in a mixture of 4.63 L of freshwater and 5.37 L of seawater; Tomita et al., [6]); Fluid 2, which has half the salinity and twice the urea concentration (prepared by dissolving 700 g of urea in a mixture of 7.31 L of freshwater and 2.69 L of seawater); Fluid 3, which has one-third the salinity and three times the urea concentration (prepared by dissolving 1050 g of urea in a mixture of 8.21 L of freshwater and 1.79 L of seawater); and Fluid 4, which has one-fourth the salinity and four times the urea concentration (prepared by dissolving 1400 g of urea in a mixture of 8.66 L of freshwater and 1.34 L of seawater).

One hour prior to the experiment, we extracted parasites, *Myxinidocotyle japonica* (Monodenea: Acanthocotylidae), from a purple hagfish (*Eptatretus okinoseanus*) maintained at Okinawa Churaumi Aquarium (Okinawa, Japan), and kept them in 8 °C seawater. Taxonomic identification of the parasite was based on methods described by Malmberg and Fernholm [7] and Vaughan and Christison [8]. The parasites were divided into four groups (21 individuals per group) and placed separately in plastic dishes filled with 8 °C Fluid 1, 2, 3, or 4. The number of live parasites in each group was counted every minute using a Leica M165C stereomicroscope (Leica Microsystems GmbH, Wetzlar, Germany).

All parasites exhibited similar behavioral changes over time after exposure to the urea water: Immediately after exposure to urea water, they expanded and contracted their entire bodies. This behavior gradually weakened and eventually stopped. After external body motion ceased, internal body movement persisted. This included rhythmic contractions of the internal organs. These internal movements slowed, weakened, and eventually ceased (defined as “death” in this study). These individuals did not recover even when returned to seawater.

**Fig. 2:**
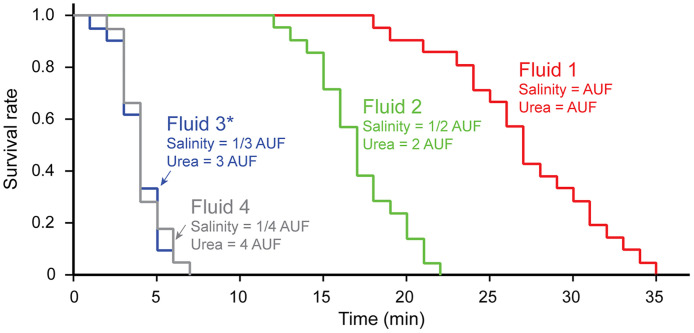
Survival curves of the monogenean parasite (*Myxinidocotyle japonica*) in urea water with different chemical compositions (Fluids 1–4). Fluid 1: Salinity and urea concentration are the same as those of the shark-specific artificial uterine fluid (AUF) described by Tomita et al. [6]. Fluid 2: Salinity is half that of AUF, and urea concentration is twice that of AUF. Fluid 3: Salinity is one-third that of AUF, and urea concentration is three times that of AUF. Fluid 4: Salinity is one-fourth that of AUF, and urea concentration is four times that of AUF. Asterisk: Fluid 3 was adopted as the standard treatment protocol for hagfish in this study.

Fig. 2 shows the survival curves of parasites in the four groups (exposed to Fluids 1–4), indicating that increasing the urea concentration and decreasing the salinity generally led to faster parasite elimination (90 % mortality occurred at 33, 20, 5, and 6 min in Fluids 1, 2, 3, and 4, respectively). Given that there was no significant difference in the mean survival time of parasites between Fluids 3 and 4 (*p* = 0.64 > 0.05, *t*-test), the use of Fluid 4—despite its more extreme salinity—offers little advantage over Fluid 3. Since purple hagfish can tolerate exposure to Fluid 3 for >5 min, we adopted a 5-minute bath in Fluid 3 as the standard treatment protocol. The optimal fluid chemistry and bathing duration may vary depending on the parasite and host species.

### Example of method application

We applied this method to three purple hagfish that were heavily infected with *Myxinidocotyle japonica*. These hagfish were originally caught in the sea near the main island of Okinawa at a depth of approximately 700 m using bait traps, and have been maintained in a tank with 8 °C seawater at Okinawa Churaumi Aquarium (Okinawa, Japan). The total lengths and weights of the individuals were 35.0, 53.5, and 55.0 cm, and 118, 309, and 454 g, respectively.

**Fig. 3:**
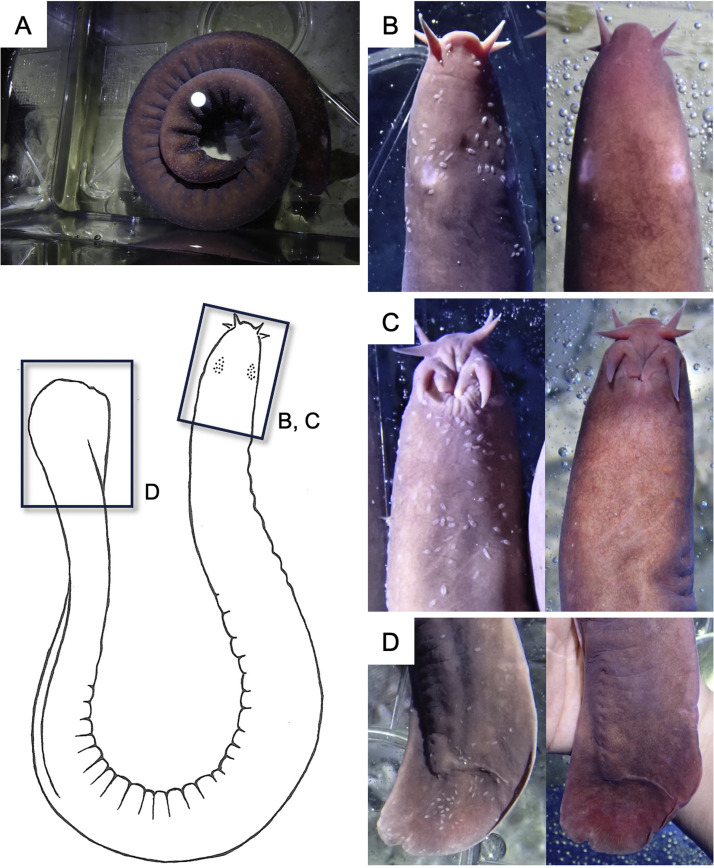
Urea-water bathing for captive purple hagfish (*Eptatretus okinoseanus*). A parasite-infected hagfish undergoing urea-water bathing (**A**). Comparisons of the skin surface before (left) and after (right) treatment: (**B**, head, dorsal view; **C**, head, ventral view; **D**, tail, lateral view). Numerous monogenean parasites (*Myxinidocotyle japonica*) visible as white particles in the photographs, covered the entire body surface before treatment but were completely removed afterward.

For the treatment, each hagfish was moved from the seawater tank to a 30-L plastic container placed next to the seawater tank (Fig. 3A). After 5 min of urea-water bathing, the fish were returned to their original seawater tanks.

After treatment, almost all visible parasites detached from the body (Fig. 3B-D) and fell to the bottom of the plastic container. Some parasites remained on the skin surface, but they died and detached easily when the husbandry staff wiped the skin by hand. During treatment, the hagfish did not exhibit strong behavioral (e.g., abnormal respiration or vigorous body undulation) or physiological responses (e.g., excessive mucous secretion). All hagfish survived for more than two months after treatment, suggesting the safety of this method.

### Comparisons to the previous methods

Commonly used methods to treat monogenean infections in aquaculture—namely freshwater baths and praziquantel administration [9,10]—are lethal to hagfish and therefore not applicable. Manual removal of parasites using forceps is time-consuming, and it is practically impossible to eliminate all parasites distributed across the entire body surface. Therefore, to the best of our knowledge, urea-water bathing is the only effective method for treating monogenean infections in hagfish.

## Limitations of the present method and future direction

The applicability of urea water as a parasiticidal bathing fluid has so far only been confirmed in hagfish and the applicability to other fish taxa still remains unknown. However, we expect that urea water baths would also be applicable as a parasiticidal to elasmobranchs (sharks and batoids), which are less tolerant of hypoosmotic environments compared to teleosts, and for which freshwater baths are not suitable. Our preliminary experiments have confirmed that the two host species of shark (*Scyliorhinus tokubee* and *Mustelus manazo*) can tolerate the 5-min exposure to urea water. Survival tests in the urea water for the monogenean parasites that are isolated from infected elasmobranchs, as demonstrated in Fig. 2, are the next step towards evaluating the applicability of the parasiticidal method evaluated in the present study.

## Ethics statements

The handing of the animal was conducted in strict accordance with the guidelines for animal experiments of the Okinawa Churashima Foundation, with the same consideration for animal care and welfare as applied to “higher” vertebrates (reptiles, birds, and mammals). As stipulated in the guidelines, approval from the Institutional Animal Care and Use Committee of Okinawa Churashima Foundation—required for higher vertebrates—is waived for “lower” vertebrates, including fishes.

## Related research article

None.

## For a published article

None.

Supplementary material *and/or* additional information [OPTIONAL]

Not applicable

## Declaration of competing interest

The authors declare that they have no known competing financial interests or personal relationships that could have appeared to influence the work reported in this paper.

## Data Availability

Data will be made available on request.
